# A systematic approach to designing statistically powerful heteroscedastic 2 × 2 factorial studies while minimizing financial costs

**DOI:** 10.1186/s12874-016-0214-3

**Published:** 2016-08-31

**Authors:** Show-Li Jan, Gwowen Shieh

**Affiliations:** 1 Department of Applied Mathematics, Chung Yuan Christian University, Taoyuan, 32023 Taiwan; 2 Department of Management Science, National Chiao Tung University, Hsinchu, 30010 Taiwan

**Keywords:** Budget, Factorial design, Heteroscedasticity, Interaction, Power, Sample size

## Abstract

**Background:**

The 2 × 2 factorial design is widely used for assessing the existence of interaction and the extent of generalizability of two factors where each factor had only two levels. Accordingly, research problems associated with the main effects and interaction effects can be analyzed with the selected linear contrasts.

**Methods:**

To correct for the potential heterogeneity of variance structure, the Welch-Satterthwaite test is commonly used as an alternative to the *t* test for detecting the substantive significance of a linear combination of mean effects. This study concerns the optimal allocation of group sizes for the Welch-Satterthwaite test in order to minimize the total cost while maintaining adequate power. The existing method suggests that the optimal ratio of sample sizes is proportional to the ratio of the population standard deviations divided by the square root of the ratio of the unit sampling costs. Instead, a systematic approach using optimization technique and screening search is presented to find the optimal solution.

**Results:**

Numerical assessments revealed that the current allocation scheme generally does not give the optimal solution. Alternatively, the suggested approaches to power and sample size calculations give accurate and superior results under various treatment and cost configurations.

**Conclusions:**

The proposed approach improves upon the current method in both its methodological soundness and overall performance. Supplementary algorithms are also developed to aid the usefulness and implementation of the recommended technique in planning 2 × 2 factorial designs.

**Electronic supplementary material:**

The online version of this article (doi:10.1186/s12874-016-0214-3) contains supplementary material, which is available to authorized users.

## Background

The factorial designs are the most common formulation for assessing the existence of interaction and the extent of generalizability of two or more factors in medical research. Notably, systematic reviews and practical guidelines have been presented in Green, Liu, and O’Sullivan [1], Kahan [2], Kent et al. [3], McAlister et al. [4], and Montgomery, Astin, and Peters [5]. The prominent advantages of factorial designs over a series of single-factor studies can be easily seen with the 2 × 2 factorial design. Particularly, a wide range of research problems associated with interactions, main effects, and various mixtures can be examined in terms of a linear combination of mean effects. It is noteworthy that the designated linear comparison represents the substantive hypothesis of interest and reveals essential information that cannot be obtained from single-factor studies. Comprehensive exposition and further information can be found in Kutner et al. [6] and Maxwell and Delaney [7]. In addition, useful flowcharts of two-factor studies were presented in Figure 19.11 of Kutner et al. [6] and Figure 7.2 of Maxwell and Delaney [7] for appropriate approaches to evaluating effects either in the presence or the absence of an interaction.

It follows from the independence, normality, and homogeneity of variance assumptions, that the inference for a linear combination of mean effects can be conducted with a *t* statistic. However, numerous studies have found that the homogeneous variances assumption is frequently untenable in many research areas. It was explicitly stressed in Golinski and Cribbie [8], Grissom [9], Keselman et al. [10], and Ruscio and Roche [11] that variances are often extremely different across treatment groups. Therefore, it is prudent to adopt proper approaches that are superior to the traditional methods under various circumstances of heterogeneous variances. For testing a hypothesis of a linear combination of group means, the approximation suggested independently by Satterthwaite [12] and Welch [13] is the most widely recommended technique to correct for variance heterogeneity (Kirk [14]; Maxwell & Delaney [7]). The procedure is sometimes referred to as the Welch–Satterthwaite test and provides a simple and robust *t*-solution with approximate degrees of freedom.

A research study typically requires adequate statistical power and sufficient sample size to detect scientifically credible effects. Within the context of medical trials, the power and sample size implications for subgroup analysis of treatment combination and interaction in factorial studies were noted in Beck et al. [15], Brookes et al. [16], Gonen [17], Natarajan et al. [18], and Wolbers et al. [19]. To extend the applicability of the Welch–Satterthwaite procedure in planning research designs, Shieh and Jan [20] presented two approaches to power and sample size calculations for the Welch–Satterthwaite test. The approximate method presents a particularly convenient technique for general use. Alternatively, the exact formulation is noticeably more effective in maintaining the power performance in some situations. However, the prescribed approaches for choosing sample size to provide adequate power do not consider the cost issues of different sample size choices. But the cost for treating a subject often varies with treatment groups and it is impossible for researchers to overlook budget constraints in practice. Bacchetti [21] and Bacchetti, McCulloch, and Segal [22] also emphasized that the conventional sample size procedures do not take cost into account and can produce cost-inefficient sample size choices. Alternatively, Allison et al. [23] advocated designing statistically powerful studies while minimizing costs.

Recently, Luh and Guo [24] studied the problem of efficient sample size allocation to reduce budget for the Welch–Satterthwaite test within the context of 2 × 2 factorial design. The optimal ratio of sample sizes suggested in Luh and Guo [24] is proportional to the ratio of the population standard deviations divided by the square root of the ratio of the unit sampling costs. Despite the presented argument and demonstration, their method is susceptible to three critical issues. First, the two-step procedure of Luh and Guo [24] involved several approximations including the use of quantiles of a standard normal distribution and a *t* distribution with approximate degrees of freedom. Unlike the two approaches of Shieh and Jan [20], Luh and Guo [24] did not employ the feature of a noncentral *t* distribution in their computation. More importantly, they did not explicitly define the nonnull distribution and the power function of the Welch–Satterthwaite test. Therefore, the resulting explication is incomplete by its absence of vital technical formulations.

Second, the optimal sample size ratios in Luh and Guo [24] were obtained with the simplified assumption that the test statistic has a normal distribution with known variances. Note that the particular formula has been adopted in Guo and Luh [25] to determine the presumably optimal sample size ratios for designing statistically powerful two-sample studies while minimizing financial costs. However, it was shown in Jan and Shieh [26] that such an allocation scheme generally do not give the optimal solution. Hence, it is arguable that Luh and Guo’s [24] claim of optimal allocations requires further clarification. Third, the sample sizes need to be integer values in reality. The final sample sizes in Luh and Guo [24] are determined by rounding up the outcomes of the two-step calculations to the next largest integers. Conceivably, the use of discrete numbers induces some inexactness into the optimal evaluation. It is unlikely that a direct integer rounding process will always give the optimal result under power and cost considerations even that the optimal sample size ratios have been implemented. Instead, a systematic power calculation and cost assessment needs to be conducted to find the proper result.

In order to make a useful and well-supported recommendation on optimal sample size allocations, this article presents an alternative approach for designing statistically powerful heteroscedastic 2 × 2 factorial studies while minimizing financial costs. A detailed account of the Welch–Satterthwaite test is presented next to document its theoretical characteristics and computational requirements. Moreover, the optimization processes of the proposed procedure with power and cost constraints are described. To provide definitive evidence, extensive empirical investigations were conducted to demonstrate the advantages of the suggested approach over the potentially defective method of Luh and Guo [24] under a variety of model configurations. Essentially, this study contributes to the literature of sample size methodology for the Welch–Satterthwaite test in two aspects. First, the suggested allocation scheme extends the design strategy of Shieh and Jan [20] to accommodate both budgetary constraints and power assessments. Second, the proposed optimization technique offers prominent improvement over Luh and Guo’s [24] method to obtain the true optimal sample sizes. For computing the attained power and optimal allocation scheme in planning research designs, the SAS computer algorithms are offered to facilitate the recommended procedure.

## Methods

Consider the statistical model of a 2 × 2 heteroscedastic factorial design:

1 $$ {X}_{ijk}\sim N\left({\mu}_{ij},\kern0.5em {\sigma}_{ij}^2\right), $$

where *X*_*ijk*_ represents the independent and normally distributed response variable with expected values μ_*ij*_ and variances σ_*ij*_^2^, μ_*ij*_ is the population mean, and σ_*ij*_^2^ is the error variance at level *i* of the factor A and level *j* of the factor B for *i* and *j* = 1 and 2, and *k* = 1, …, *N*_*ij*_. In general, a linear combination of mean parameters is defined as

2 $$ \uppsi ={\displaystyle \sum_{i=1}^2{\displaystyle \sum_{j=1}^2{L}_{ij}{\upmu}_{ij}}} $$

where *L*_*ij*_ are the linear coefficients. A contrast is a special case of a linear combination in which the coefficients of the means add up to 0. Notably, the contrasts associated with the main effect A, the main effect B, and the interaction effect between A and B can be expressed as

$$ \begin{array}{l}{\uppsi}_A={\upmu}_{11}+{\upmu}_{12}\hbox{--} {\upmu}_{21}\hbox{--} {\upmu}_{22},\\ {}{\uppsi}_B={\upmu}_{11}\hbox{--} {\upmu}_{12}+{\upmu}_{21}\hbox{--} {\upmu}_{22},\end{array} $$

and

3 $$ {\uppsi}_I={\upmu}_{11}\hbox{--} {\upmu}_{12}\hbox{--} {\upmu}_{21}+{\upmu}_{22}, $$

respectively. An unbiased estimator $\widehat{\uppsi}$ of the linear combination ψ is obtained by simply replacing each population mean in Equation 2 by the corresponding sample mean:

4 $$ \widehat{\uppsi}={\displaystyle \sum_{i=1}^2{\displaystyle \sum_{j=1}^2{L}_{ij}{\overline{X}}_{ij},}} $$

where ${\overline{X}}_{ij}={\displaystyle \sum_{k=1}^{N_{ij}}{X}_{ijk}}/{N}_{ij}$ for *i* and *j* = 1 and 2. The hypothesis testing of H_0_: ψ = ψ_0_ versus H_1_: ψ ≠ ψ_0_ can be conducted with the following statistic

5 $$ T*=\frac{\widehat{\uppsi}-{\uppsi}_0}{\widehat{\upomega}}, $$

where ψ_0_ is a specified constant, ${\widehat{\upomega}}^2={\displaystyle \sum_{i=1}^2{\displaystyle \sum_{j=1}^2{L}_{ij}^2{S}_{ij}^2}}/{N}_{ij}$ is the typical estimator of ${\upomega}_{\cdot}^2=Var\left(\widehat{\uppsi}\right)={\displaystyle \sum_{i=1}^2{\displaystyle \sum_{j=1}^2{L}_{ij}^2{\sigma}_{ij}^2}}/{N}_{ij}$, and ${S}_{ij}^2={\displaystyle \sum_{l=1}^{Nij}{\left({X}_{ijk}-{\overline{X}}_{ij}\right)}^2}/\left({N}_{ij}-1\right)$ is the sample variance estimator of σ_*ij*_^2^ for *i* and *j* = 1 and 2. Under the null hypothesis H_0_: ψ = ψ_0_, it was demonstrated in Satterthwaite [12] and Welch [13] that the statistic T* given in Equation 5 has a convenient approximate distribution

6 $$ T*\overset{.}{\sim }t(v), $$

where *t*(ν) is a *t* distribution with degrees of freedom ν and

$$ v={\left\{{\displaystyle \sum_{i=1}^2{\displaystyle \sum_{j=1}^2{L}_{ij}^2{\upsigma}_{ij}^2}}/{N}_{ij}\right\}}^2/\left\{{\displaystyle \sum_{i=1}^2{\displaystyle \sum_{j=1}^2{L}_{ij}^4{\upsigma}_{ij}^4}}/\left[{N}_{ij}^2\left({N}_{ij}-1\right)\right]\right\}. $$

For inferential purposes, the term of degrees of freedom ν is replaced by its counterpart $\widehat{v}$ with direct substitution of *S*_*ij*_^2^ for σ_*ij*_^2^ in ν, where

7 $$ \widehat{v}={\left\{{\displaystyle \sum_{i=1}^2{\displaystyle \sum_{j=1}^2{L}_{ij}^2}}{S}_{ij}^2/{N}_{ij}\right\}}^2/\left\{{\displaystyle \sum_{i=1}^2{\displaystyle \sum_{j=1}^2{L}_{ij}^4{S}_{ij}^4}}/\left[{N}_{ij}^2\left({N}_{ij}-1\right)\right]\right\}. $$

Hence, the null distribution of T* is modified as

8 $$ T*\overset{.}{\sim }t\left(\widehat{v}\right), $$

and the Welch–Satterthwaite procedure rejects H_0_ at the significance level α if $\left|T*\right|>{t}_{\widehat{v},a/ 2}$ where ${t}_{\widehat{v},a/2}$ is the upper 100(α/2) percentile of the *t* distribution $t\left(\widehat{v}\right)$.

Moreover, it was noted in Shieh and Jan [20] that the statistic T* has the general approximate distribution

9 $$ T*\overset{.}{\sim }t\left(v,\kern0.5em \updelta \right), $$

where *t*(ν, δ) is a noncentral *t* distribution with degrees of freedom ν and noncentrality parameter

$$ \updelta =\frac{\uppsi -{\uppsi}_0}{\upomega}. $$

It immediately follows from the noncentral *t* distribution given in Equation 9 that the power function of the Welch–Satterthwaite test can be approximated by

10 $$ \uppi \left(\updelta \right)=P\left\{\left|t\left(v,\updelta \right)\right|>{t}_{v,a/2}\right\}. $$

Accordingly, Shieh and Jan [20] noted that the approximate power function π(δ) provides a useful expression because of its theoretical implications and practical applications. The numerical computation of the power level requires the evaluation of the cumulative distribution function of a noncentral *t* variable with respective to the quantile of a regular *t* distribution. Since all related functions are embedded in major statistical packages, the actual computations can be readily conducted with current computing capabilities. A SAS/IML (SAS Institute [27]) program is presented in Additional file 1 for computing π(δ) with the designated sample sizes and model configurations. More importantly, the empirical examinations presented later reveal that the approximate power function is sufficiently accurate for general purposes.

### Optimal allocation scheme

The determination of an adequate and efficient allocation of sample sizes is a vital aspect in the planning stage of research studies. It is often sensible to consider the sample size issues in the presence of funding constraints. The total cost of a 2 × 2 factorial study can be represented by the overhead cost and sampling costs through the following linear cost function

11 $$ {C}_T={C}_o+{\displaystyle \sum_{i=1}^2{\displaystyle \sum_{j=1}^2{C}_{ij}{N}_{ij},}} $$

where *C*_*O*_ is the fixed overhead cost associated with the study and *C*_*ij*_ reflects unit sampling cost of each subject in group (*i*, *j*) for *i* and *j* = 1 and 2. It is important to note that the consideration of the total number of subjects can be viewed as a special case of the cost function *C*_*T*_, with *C*_*O*_ = 0 and *C*_*ij*_ = 1 for *i* and *j* = 1 and 2. From the cost perspective, a question arises naturally in choosing the optimal sample sizes: What is the least cost for a research study to maintain its desired power level?

To develop a systematic search of the optimal solution to ensure the nominal power performance while minimizing the total cost *C*_*T*_ defined in Equation 11, the suggested approach is conducted in two steps. With the specifications of the significance level α, the desired power level 1 – β, the null effect size ψ_0_, and the model parameters of group means and variance components, the first step computes the preliminary sample sizes {*N*_*P*11_, *N*_*P*12_, *N*_*P*21_, *N*_*P*22_} via an optimization process by minimizing the objective cost function with the constraint that the attained power is equal to or greater than the designated level. Note that the attained power is computed with the approximate power function π(δ) of the Welch–Satterthwaite test defined in Equation 10. A closed form solution rarely exists for most situations and therefore the minimization typically requires iterative and extensive computations.

Accordingly, the NLPQN subroutine of the SAS/IML package provides an efficient approach to finding the optimal solution for cost minimization with the power function as a nonlinear constraint. It must be emphasized that the sample sizes are treated as continuous variables in the optimization process. Thus, the resulting values {*N*_*P*11_, *N*_*P*12_, *N*_*P*21_, *N*_*P*22_} are almost surely not all integers. Due to the underlying metric of integer sample sizes, in practice, the values are rounded up to the nearest integer. This simple and intuitive adjustment maintains that the corresponding power is still no less than the nominal power. But both the achieved power and total cost actually increase for the modified sample sizes {*N*_*M*11_, *N*_*M*12_, *N*_*M*21_, *N*_*M*22_} = {[*N*_*P*11_] + 1, [*N*_*P*12_] + 1, [*N*_*P*21_] + 1, [*N*_*P*22_] + 1}, where [*N*] denotes the integer part of *N*. Notably, the optimal property of the sample sizes {*N*_*P*11_, *N*_*P*12_, *N*_*P*21_, *N*_*P*22_} does not necessarily carry over to the adjusted sample sizes {*N*_*M*11_, *N*_*M*12_, *N*_*M*21_, *N*_*M*22_}. In sum, the rounding process tends to induce a suboptimal solution and the optimal set of sample sizes remains to be determined.

In the second step, a detailed comparison is conducted to find the proper result by taking into account the discrete character of sample sizes in practice. Specifically, power calculations and cost evaluations are performed for a total of 2^4^ = 16 sample size sets {*N*_11_, *N*_12_, *N*_21_, *N*_22_} with *N*_*ij*_ = [*N*_*Pij*_] or [*N*_*Pij*_] + 1 for *i* and *j* = 1 and 2. Then the optimal allocation {*N*_11_^*^, *N*_12_^*^, *N*_21_^*^, *N*_22_^*^} is found through a screening of the sample size combinations that attain the desired power while giving the least cost. If more than one combination yields the same magnitude of least cost, the one producing the largest power is reported. Note that this fine-tuning procedure can be considered as a safeguard to ensure that the nearly optimal and integer sample sizes {*N*_*M*11_, *N*_*M*12_, *N*_*M*21_, *N*_*M*22_} is the true optimal solution. Unfortunately, the conducted numerical calculations revealed that the sample sizes {*N*_*M*11_, *N*_*M*12_, *N*_*M*21_, *N*_*M*22_} are rarely the correct optimal allocation. This finding justifies the suggested screening technique and notifies the deficiency of the rounding process in Luh and Guo [24]. A special purpose SAS/IML computer program is presented in Additional file 2 for performing the necessary computation.

On the other hand, Luh and Guo [24] presented a two-step procedure for obtaining the optimal result. First, using the simplified normal assumption, they showed that the optimal sample size ratio is proportional to the ratio of the population standard deviations divided by the square root of the ratio of the unit sampling costs:

12 $$ {r}_{ij}=\frac{N_{ij}}{N_{11}}=\frac{\upsigma_{ij}{C}_{11}^{1/2}}{\upsigma_{11}{C}_{ij}^{1/2}}, $$

*i* and *j* = 1 and 2. The initial sample sizes {*N*_*Z*11_, *N*_*Z*12_, *N*_*Z*21_, *N*_*Z*22_} are obtained with *N*_*Zij*_ = *N*_*Z*11·_*r*_*ij*_, *i* and *j* = 1 and 2, where

$$ {N}_{Z11}=\frac{{\left({Z}_{\alpha /2}+Z\upbeta \right)}^2{w}^2}{{\left(\uppsi -{\uppsi}_0\right)}^2}, $$

${w}^2={\displaystyle \sum_{i=1}^2{\displaystyle \sum_{j=1}^2{L}_{ij}^2{\upsigma}_{ij}^2}}/{r}_{ij},$ and *z*α_/2_ and *z*β are the upper 100(α/2)th and 100 · βth percentiles of the standard normal distribution, respectively. Then, to account for the approximate degrees of freedom of the Welch–Satterthwaite test, they suggested a modified process by using the sample sizes {*N*_*Z*11_, *N*_*Z*12_, *N*_*Z*21_, *N*_*Z*22_} to yield the second set of sample sizes {*N*_*T*11_, *N*_*T*12_, *N*_*T*21_, *N*_*T*22_} where *N*_*Tij*_ = *N*_*T*11·_*r*_*ij*_, *i* and *j* = 1 and 2,

$$ {N}_{T11}=\frac{{\left({t}_{\upsilon, {}^{\circ}\alpha /2}+{t}_{\upsilon, {}^{\circ}\upbeta}\right)}^2{w}^2}{{\left(\uppsi -{\uppsi}_0\right)}^2}, $$

and

$$ \upsilon ={\left\{{\displaystyle \sum_{i=1}^2{\displaystyle \sum_{j=1}^2{L}_{ij}^2{\upsigma}_{ij}^2}}/{N}_{Zij}\right\}}^2/\left\{{\displaystyle \sum_{i=1}^2{\displaystyle \sum_{j=1}^2{L}_{ij}^4{\upsigma}_{ij}^4}}/\left[{N}_{Zij}^2\left({N}_{Zij}-1\right)\right]\right\}. $$

Because the values of {*N*_*T*11_, *N*_*T*12_, *N*_*T*21_, *N*_*T*22_} are most likely fractional, a direct rounding process is applied to give the final sample sizes {*N*_*LG*11_, *N*_*LG*12_, *N*_*LG*21_, *N*_*LG*22_} where *N*_*LGij*_ = [*N*_*Tij*_] + 1 for *i* and *j* = 1 and 2. In contrast to the proposed fine-tuning algorithm, Luh and Guo [24] overlooked the inexactness issue caused by integer sample sizes in power evaluation and cost minimization. This is one of possible causes that their method does not guarantee to produce the optimal sample sizes as shown in the subsequent numerical comparisons.

### Numerical assessments

To illustrate the advantage of the proposed optimal procedure over the existing method of Luh and Guo [24], numerical appraisals were performed to assess the optimal sample size calculations of the two methods under a wide variety of model configurations. The empirical investigation consists of two studies with real and hypothetical data that correspond to the model settings in Tables 2 and 3 of Luh and Guo [24].

#### Study I

For the purposes of comparison, the illustrative example for the 2 × 2 factorial study of attack context and panic fear in Luh and Guo [24] is reexamined here. The data was obtained from the investigations of frequency and cost of emergency service use in Barnett and Nurmagambetov [28] and Greaves et al. [29]. To exemplify a typical research scenario most frequently encountered in the planning stage of a study, the reported findings are employed to provide planning values of the model parameters and design characteristics for future asthma study.

Specifically, the mean effects and variance components are designated as {μ_11_, μ_12_, μ_21_, μ_22_} = {1.23, 0.42, 0.13, 0.38} and {σ_11_^2^, σ_12_^2^, σ_21_^2^, σ_22_^2^} = {0.6889, 0.5184, 0.1156, 0.5929}, respectively. The contrast effect sizes associated with the main effect A (Asthma attack: recent and stable), the main effect B (Panic fear: low and high), and the interaction effect defined in Equation 3 are ψ_*I*_ = 1.06, ψ_*A*_ = 1.14, and ψ_*B*_ = 0.56, respectively. For the definition of total cost, the fixed cost is set as *C*_*O*_ = 0, and two sets of unit costs *C*_*U*_ = {784.74, 267.96, 82.94, 242.44} and *C*_*E*_ = {1, 1, 1, 1} are considered to represent varied and identical unit sampling costs for the four treatment groups. Then the proposed allocation procedure was employed to find the optimal sample sizes needed to achieve the nominal power 1 – β = 0.8 for three contrast effects and two cost structures. Throughout this empirical study, the significance level is set as α = 0.05 and the null value is ψ_0_ = 0. Overall, a total of six different sets of sample sizes were obtained.

The optimal allocation, total cost, and total sample size are summarized in Table 1. Unlike the presented procedure, the numerical outcomes reported in Table 2 of Luh and Guo [24] are based on the sample size ratios in Equation 12. For ease of illustration, the corresponding results are also presented in Table 1. In addition, the attained powers for the computed sample sizes were computed by the suggested approximate power function π(δ) given in Equation 10. Due to the approximate nature of the suggested power calculations, Monte Carlo simulation of 10,000 independent data sets was also conducted to obtain the simulated powers. Accordingly, the adequacy of the approximate power function can be evaluated by the difference between the simulated power and approximate power. In addition to the prescribed optimal sample allocation, total cot and total sample size, the approximate power, simulated power and difference are also listed in Table 1.

**Table 1:** Computed sample size, total cost, total size, simulated power, and error for the approaches of the proposed approach and Luh and Guo’s (2016) method, when α = 0.05, 1 – β = 0.8, and {σ_11_^2^, σ_12_^2^, σ_21_^2^, σ_22_^2^} = (0.6889, 0.5184, 0.1156, 0.5929)

ψ^*a*^	Unit costs^*b*^	Method	Sample sizes	Total cost	Total sample size	Approximate power^*c*^	Simulated power	Error
ψ_*I*_	C_*U*_	Proposed procedure	{11, 16, 13, 19}	18604.08	59	0.8005	0.7964	0.0041
		Luh and Guo	{12, 17, 14, 19}	19739.72	62	0.8254	0.8169	0.0085
	C_*E*_	Proposed procedure	{16, 14, 7, 15}	52	52	0.8038	0.8053	–0.0015
		Luh and Guo	{17, 14, 7, 15}	53	53	0.8113	0.8077	0.0036
ψ_*A*_	C_*U*_	Proposed procedure	{10, 13, 12, 16}	16205.20	51	0.8004	0.7906	0.0098
		Luh and Guo	{10, 15, 13, 17}	17066.50	55	0.8208	0.8157	0.0051
	C_*E*_	Proposed procedure	{14, 12, 6, 13}	45	45	0.8014	0.8012	0.0002
		Luh and Guo	{15, 13, 6, 14}	48	48	0.8273	0.8304	–0.0031
ψ_*B*_	C_*U*_	Proposed procedure	{38, 56, 48, 62}	63838.28	204	0.8000	0.7889	0.0111
		Luh and Guo	{38, 57, 48, 64}	64591.12	207	0.8046	0.8019	0.0027
	C_*E*_	Proposed procedure	{56, 49, 23, 52}	180	180	0.8021	0.7969	0.0052
		Luh and Guo	{56, 49, 23, 52}	180	180	0.8021	0.8008	0.0013

Note: ^*a*^The contrast effects are ψ_*I*_ = 1.06, ψ_*R*_ = 1.14, and ψ_*C*_ = 0.56. ^*b*^The cost coefficients are *C*_*U*_ = {784.74, 267.96, 82.94, 242.44} and *C*_*E*_ = {1, 1, 1, 1}. ^*c*^The attained power computed by the suggested approximate power function

**Table 2:** Computed sample size, total cost, total size, simulated power, and error for the approaches of the proposed approach and Luh and Guo’s (2016) method, when α = 0.05, 1 – β = 0.8, ψ_*I*_ = 2, and {σ_11_^2^, σ_12_^2^, σ_21_^2^, σ_22_^2^} = (1, 4, 9, 16)

Unit costs	Method	Sample sizes	Total cost	Total sample size	Approximate power^*a*^	Simulated power	Error
{1, 1, 1, 1}	Proposed procedure	{20, 40, 60, 79}	199	199	0.8016	0.8002	0.0014
	Luh and Guo	{20, 40, 60, 80}	200	200	0.8036	0.8029	0.0007
{1, 2, 3, 4}	Proposed procedure	{33, 48, 58, 68}	575	207	0.8000	0.7971	0.0029
	Luh and Guo	{34, 48, 59, 68}	579	209	0.8028	0.8050	–0.0022
{4, 3, 2, 1}	Proposed procedure	{14, 32, 57, 108}	374	211	0.8009	0.7979	0.0030
	Luh and Guo	{14, 32, 58, 109}	377	213	0.8041	0.8047	–0.0006
{1, 1, 2, 5}	Proposed procedure	{32, 63, 68, 58}	521	221	0.8001	0.7928	0.0073
	Luh and Guo	{33, 65, 69, 58}	526	225	0.8038	0.8033	0.0005
{5, 2, 1, 1}	Proposed procedure	{11, 34, 72, 95}	290	212	0.8006	0.8000	0.0006
	Luh and Guo	{11, 34, 73, 97}	293	215	0.8046	0.8089	–0.0043
{1, 3, 3, 1}	Proposed procedure	{27, 32, 47, 107}	371	213	0.8004	0.8030	–0.0026
	Luh and Guo	{28, 32, 48, 109}	377	217	0.8039	0.8067	–0.0028

^*a*^The attained power computed by the suggested approximate power function

#### Study II

To further explicate the optimal behavior and profound implication of the two sample size procedures, additional numerical assessments were performed with different variability patterns and cost structures. In this study, the simulation design of Luh and Guo [24] is adopted as a convenient framework. The model configurations are {μ_11_, μ_12_, μ_21_, μ_22_} = {1, 0, 0, 1} and {σ_11_^2^, σ_12_^2^, σ_21_^2^, σ_22_^2^} = {1, 4, 9, 16}. Here, the focus is on the detection of the interaction effect ψ_*I*_ = μ_11_ – μ_12_ – μ_21_ + μ_22_ = 2. For the calculation of total cost, the fixed cost is set as *C*_*O*_ = 0, and six sets of unit costs are considered: {*C*_11_, *C*_12_, *C*_21_, *C*_22_} = {1, 1, 1, 1}, {1, 2, 3, 4}, {4, 3, 2, 1}, {1, 1, 2, 5}, {5, 2, 1, 1}, and {1, 3, 3, 1}. These combinations of unit cost patterns were chosen to represent as much as possible the extent of characteristics that are likely to be obtained in actual applications. Similar to the illustration in Study I, the main settings are assigned as the significance level α = 0.05, nominal power 1 – β = 0.8, and the null value ψ_0_ = 0. Also, Monte Carlo simulation of 10,000 independent data sets was again conducted to obtain the simulated powers. The computed sample sizes, total cost, total sample size, approximate power, simulated power, and associated error of the two competing approaches are presented in Table 2 for six different cost functions.

## Results

As was pointed out above, Luh and Guo [24] did not explicitly describe the power function for the Welch–Satterthwaite test and only the simulated powers were reported in their numerical demonstration. Note that the approximate powers presented in Table 1 of both sample size procedures are computed with respect to the approximate power function π(δ) for the reported sample sizes. The appraisal and implication of power performance are valid only when the power function provides reasonably accurate results. With the target power 0.8, it is evident that all the errors between the approximate power and simulated power are contained in the interval [–0.0031, 0.0111]. Hence, the presented power function in Equation 10 and resulting powers appear to be accurate enough to validate optimization analysis. Moreover, all the approximate powers induced by the computed sample sizes are greater than the nominal level 0.8 for all 12 cases. Hence, the two contending optimal allocations provide adequate power to validate further methodological comparisons of corresponding sample size procedures.

It can be readily seen from the reported sample sizes in Table 1 that the two allocation procedures do not agree with the optimal sample size settings. The computed sample sizes of Luh and Guo’s [24] method are larger than or equal to those of the suggested technique. The last scenario associated with ψ_*B*_ and *C*_*E*_ is the only one case where the two sets of sample sizes are identical. Specifically, the sample sizes associated with the interaction effect ψ_*I*_ and cost coefficient set *C*_*E*_ are {11, 16, 13, 19} and {12, 17, 14, 19} for the proposed approach and Luh and Guo’s [24] method, respectively. In this case, the maximum difference of cell sample sizes between the two procedures is only one. This indicates that a simple rounding process may distort an optimal solution even the sample size ratios {*r*_11_, *r*_12_, *r*_21_, *r*_22_} provide a nearly optimal result under normal approximation. Moreover, for the main effect ψ_*A*_ and cost coefficient set *C*_*U*_, the computed sample sizes for the two contending procedures are {10, 13, 12, 16} and {10, 15, 13, 17}, respectively. Hence, the cell sample sizes incur the largest difference of two units. The same situation also occurred with the setup of the main effect ψ_*B*_ and cost coefficient set *C*_*U*_. In both scenarios, the discrepancy of more than one unit in sample size determinations reveals that the normality-based sample size ratios *r*_*ij*_ given in Equation 12 is also responsible for the suboptimal behavior of Luh and Guo’s [24] method. Consequently, the total cost incurred by the proposed approach is always no larger than that of the Luh and Guo [24] procedure. These numerical evidences showed that their method does not warrant optimal sample size allocation for minimizing the total cost.

With the different variability patterns and cost structures in the second empirical study, it is clear from the marginal differences between the approximate power and simulated power in Table 2 that the approximate power function π(δ) maintains a accurate solution for power calculations. Specifically, all the absolute errors are less than 0.01 for all 12 cases. Just as in the preceding study, all the approximate powers or attained powers associated with both optimal allocation methods satisfy the desired power performance 0.8 for all six cost structures. However, the computed sample sizes in Table 2 indicate that Luh and Guo’s [24] method consistently give greater total cost and larger total sample size than the suggested technique. Notably, the accuracy of their method deteriorates as the variability of the unit costs increases. For the identical unit costs {1, 1, 1, 1}, the computed sample sizes {20, 40, 60, 79} and {20, 40, 60, 80} of the two approaches are nearly the same. However, the corresponding optimal sample sizes and total costs become prominently different when the unit costs are {1, 1, 2, 5}, {5, 2, 1, 1}, and {1, 3, 3, 1}. Accordingly, the performance of the existing method of Luh and Guo [24] is sensitive to model settings and cost schemes. In view of the potentially diverse treatment and cost configurations in factorial designs, their formula does not serve as a robust procedure for general use. Consequently, the allegedly optimal sample sizes calculations of Luh and Guo [24] are actually suboptimal, and their claim of developing the most efficient allocation for heteroscedastic 2 × 2 factorial designs is incorrect.

## Discussion

The 2 × 2 factorial design is widely used in different fields of research for assessing the interaction between two factors. However, violation of the homogeneity of variance assumption has been the target of criticism in applications of standard factorial ANOVA. For testing a hypothesis of a linear combination of group means, the Welch–Satterthwaite procedure emerges as a robust alternative to heteroscedasticity when distributions are normal. For the ultimate aim of selecting the optimal sample size allocation, the analytical argument and empirical performance of an optimization technique must be well examined before it can be adopted as a general methodology in practice. The large sample theory shows that in order to ensure the nominal power while minimizing total cost, an optimal ratio of sample sizes is proportional to the ratio of the population standard deviations divided by the square root of the ratio of the unit sampling costs. At first sight, Luh and Guo’s [24] sample size procedure is easy to use and seem to give practically useful results. However, it is unlikely that a direct implementation of the simple allocation formula with a simple integer rounding will give the optimal solution under cost considerations. Therefore, there is a need to provide a systematic and detailed process to calculate the final optimal sample sizes. Evidently, the proposed procedure and the current method of Luh and Guo [24] are prominently different in fundamental principles and demand varying computational efforts. Due to the complexity of the sample size optimization problem under power and cost considerations, a complete analytic examination is not feasible. Instead, numerical assessments were conducted to examine their unique feature and underlying discrepancy in order to better understand the selection of an appropriate approach for optimal sample size determination in 2 × 2 factorial studies. Detailed appraisals showed that Luh and Guo’s [24] procedure generally do not give the optimal solution. Alternatively, the described approach provides a superior solution for optimal sample size allocation.

## Conclusions

To enhance the usefulness of the Welch–Satterthwaite procedure in planning research designs, this article addresses the corresponding problem of designing statistically powerful heteroscedastic 2 × 2 factorial studies while minimizing financial costs. The suggested approach outperforms the current method in both its methodological soundness and overall performance. The presented sample size optimization methodology can be useful for the advocated practice of planning research design under both power and cost considerations. Computer algorithms are also developed to facilitate the implementation of the recommended power and sample size calculations in planning 2 × 2 factorial designs.

## Additional files

Additional file 1: SAS IML program for computing the attained power for Welch-Satterthwaite’s test. (DOC 12 kb) Additional file 2: SAS IML program for computing the optimal sample sizes for Welch-Satterthwaite’s test to meet a designated power level with the least cost. (DOC 27 kb)

## Abbreviations

NLPQN: The nonlinear optimization by quasi-Newton method
SAS/IML: The interactive matrix language software of SAS

## References

[1] Green S, Liu PY, O’Sullivan J (2002). Factorial design considerations. J Clin Oncol.

[2] Kahan BC (2013). Bias in randomised factorial trials. Stat Med.

[3] Kent DM, Rothwell PM, Ioannidis JP, Altman DG, Hayward RA (2010). Assessing and reporting heterogeneity in treatment effects in clinical trials: a proposal. Trials.

[4] McAlister FA, Straus SE, Sackett DL, Altman DG (2003). Analysis and reporting of factorial trials: A systematic review. J Am Med Assoc.

[5] Montgomery AA, Astin MP, Peters TJ (2011). Reporting of factorial trials of complex interventions in community settings: A systematic review. Trials.

[6] Kutner MH, Nachtsheim CJ, Neter J, Li W (2005). Applied linear statistical models.

[7] Maxwell SE, Delaney HD (2004). Designing experiments and analyzing data: A model comparison perspective.

[8] Golinski C, Cribbie RA (2009). The expanding role of quantitative methodologists in advancing psychology. Can Psychol.

[9] Grissom RJ (2000). Heterogeneity of variance in clinical data. J Consult Clin Psychol.

[10] Keselman HJ, Huberty CJ, Lix LM, Olejnik S, Cribbie R, Donahue B, Kowalchuk RK, Lowman LL, Petoskey MD, Keselman JC, Levin JR (1998). Statistical practices of educational researchers: An analysis of their ANOVA, MANOVA, and ANCOVA analyses. Rev Educ Res.

[11] Ruscio J, Roche B (2012). Variance heterogeneity in published psychological research: A review and a new index. Methodology.

[12] Satterthwaite FE (1946). An approximate distribution of estimate of variance components. Biom Bull.

[13] Welch BL (1947). The generalization of Students’ problem when several different population variances are involved. Biometrika.

[14] Kirk RE (2013). Experimental Design: Procedures for the behavioral sciences (4th ed.).

[15] Beck GJ, Berg RL, Coggins CH, Gassman JJ, Hunsicker LG, Schluchter MD, Williams GW (1991). Design and statistical issues of the Modification of Diet in Renal Disease Trial. The Modification of Diet in Renal Disease Study Group. Control Clin Trials.

[16] Brookes ST, Whitely E, Egger M, Smith GD, Mulheran PA, Peters TJ (2004). Subgroup analyses in randomized trials: Risks of subgroup-specific analyses; power and sample size for the interaction test. J Clin Epidemiol.

[17] Gonen M (2003). Planning for subgroup analysis: a case study of treatment-marker interaction in metastatic colorectal cancer. Control Clin Trials.

[18] Natarajan R, Turnbull BW, Slate EH, Clark LC (1996). A computer program for sample size and power calculations in the design of multi-arm and factorial clinical trials with survival time endpoints. Comput Methods Prog Biomed.

[19] Wolbers M, Heemskerk D, Chau TT, Yen NT, Caws M, Farrar J, Day J (2011). Sample size requirements for separating out the effects of combination treatments: Randomised controlled trials of combination therapy vs. standard treatment compared to factorial designs for patients with tuberculous meningitis. Trials.

[20] Shieh G, Jan SL (2015). Power and sample size calculations for testing linear combinations of group means under variance heterogeneity with applications to meta and moderation analyses. Psicológica.

[21] Bacchetti P (2010). Current sample size conventions: Flaws, harms, and alternatives. BMC Med.

[22] Bacchetti P, McCulloch CE, Segal MR (2008). Simple, defensible sample sizes based on cost efficiency. Biometrics.

[23] Allison DB, Allison RL, Faith MS, Paultre F, Pi-Sunyer X (1997). Power and money: Designing statistically powerful studies while minimizing financial costs. Psychol Methods.

[24] Luh WM, Guo JH (2016). Allocating sample sizes to reduce budget for fixed-effect 2 × 2 heterogeneous analysis of variance. J Exp Educ.

[25] Guo JH, Luh WM (2009). Optimum sample size allocation to minimize cost or maximize power for the two-sample trimmed mean test. Br J Math Stat Psychol.

[26] Jan SL, Shieh G (2011). Optimal sample sizes for Welch’s test under various allocation and cost considerations. Behav Res Methods.

[27] Institute SAS (2013). SAS/IML User’s Guide, Version 9.3.

[28] Barnett SBL, Nurmagambetov TA (2010). Costs of asthma in the United States: 2002–2007. J Allergy Clin Immunol.

[29] Greaves CJ, Eiser C, Seamark D, Halpin DMG (2002). Attack context: An important mediator of the relationship between psychological status and asthma outcomes. Thorax.

