# Supplementary material for: A systematic approach to designing statistically powerful heteroscedastic 2 × 2 factorial studies while minimizing financial costs
Source: BMC Med Res Methodol. 2016 Aug 31;16(1):114. doi: 10.1186/s12874-016-0214-3 (PMC5006374; doi:10.1186/s12874-016-0214-3)
Supplement: Additional file 1: — SAS IML program for computing the attained power for Welch-Satterthwaite’s test. (DOC 12 kb) [file 12874_2016_214_MOESM1_ESM.doc]

Additional file 1

SAS IML program for computing the attained power for Welch-Satterthwaite’s test

PROC IML;*POWER CALCULATION;

*USER SPECIFICATIONS;

*DEGNATED POWER; POWER=0.80;

*TYPE I ERROR; ALPHA=0.05;

*SAMPLE SIZES; NVEC={12 17 14 19};

*GROUP MEANS; MUVEC={1.23 0.42 0.13 0.38};

*GROUP STANDARD DEVIATION;STDVEC={0.83 0.72 0.34 0.77};

*CONTRAST; *INTERACTION;LVEC={1 -1 -1 1};

*ROW;*LVEC={1 1 -1 -1};

*COLUMN;*LVEC={1 -1 1 -1};

*END OF SPECIFICATIONS;

G=NCOL(NVEC);NT=SUM(NVEC);PSI=LVEC*MUVEC`;VARVEC=STDVEC##2;

VARPSI=(LVEC##2)*(VARVEC/NVEC)`;

DELTA=PSI/SQRT(VARPSI);DF=NT-G;DFVEC=NVEC-1;

KV=(LVEC##2)#VARVEC/NVEC;V1=SUM(KV)##2;

V2=SUM((KV##2)/(NVEC-1));DFAP=V1/V2;CRIT=TINV(1-ALPHA/2,DFAP);

APOWER=CDF('T',-CRIT,DFAP,DELTA)+SDF('T',CRIT,DFAP,DELTA);

PRINT ALPHA POWER;PRINT NVEC;PRINT MUVEC;PRINT VARVEC;

PRINT LVEC APOWER[FORMAT=8.4];

QUIT;
