# Supplementary material for: A systematic approach to designing statistically powerful heteroscedastic 2 × 2 factorial studies while minimizing financial costs
Source: BMC Med Res Methodol. 2016 Aug 31;16(1):114. doi: 10.1186/s12874-016-0214-3 (PMC5006374; doi:10.1186/s12874-016-0214-3)
Supplement: Additional file 2: — SAS IML program for computing the optimal sample sizes for Welch-Satterthwaite’s test to meet a designated power level with the least cost. (DOC 27 kb) [file 12874_2016_214_MOESM2_ESM.doc]

Additional file 2

SAS IML program for computing the optimal sample sizes for Welch-Satterthwaite’s test to meet a designated power level with the least cost

PROC IML;*OPTIMAL SAMPLE SIZE CALCULATION;

*USER SPECIFICATIONS;

*DEGNATED POWER; POWER=0.80;

*TYPE I ERROR; ALPHA=0.05;

*GROUP MEANS; MUVEC={1.23 0.42 0.13 0.38};

*GROUP STANDARD DEVIATION;STDVEC={0.83 0.72 0.34 0.77};

*UNIT COSTS;CVEC={784.74 267.96 82.94 242.44};*CVEC={1 1 1 1};

*CONTRAST;*INTERACTION;LVEC={1 -1 -1 1};

*ROW;*LVEC={1 1 -1 -1};*COLUMN;*LVEC={1 -1 1 -1};

*END OF SPECIFICATIONS;

PRINT ALPHA POWER;PRINT MUVEC;PRINT STDVEC;PRINT LVEC;PRINT CVEC;

START C(NVEC) GLOBAL(CVEC);

TC=CVEC*NVEC`;

RETURN(TC);FINISH;

START P(NVEC) GLOBAL(G, VARVEC, MUVEC, LVEC, ALPHA, POWER);

NT=SUM(NVEC);PSI=LVEC*MUVEC`;

VARPSI=(LVEC##2)*(VARVEC/NVEC)`;

DELTA=PSI/SQRT(VARPSI);DF=NT-G;DFVEC=NVEC-1;

KV=(LVEC##2)#VARVEC/NVEC;V1=SUM(KV)##2;

V2=SUM((KV##2)/(NVEC-1));DFAP=V1/V2;CRIT=TINV(1-ALPHA/2,DFAP);

APP=CDF('T',-CRIT,DFAP,DELTA)+SDF('T',CRIT,DFAP,DELTA);

P=POWER-APP;

RETURN(P);FINISH;

G=NCOL(MUVEC);VARVEC=STDVEC##2;

OPTN=J(1,11,.);OPTN[1]=0;OPTN[2]=0;OPTN[10]=1;OPTN[11]=1;

MINNVEC=J(1,G,3);

BLC=(MINNVEC||{. .})//J(1,G+2,.);

CALL NLPQN(RC,XR,"C",MINNVEC,OPTN,BLC) NLC="P";

A={0,1};M=J(2,G,1);MAT=J(2##G,G,0);

DO I=1 TO G;MT=M;MT[,I]=A;Z=1;DO J=1 TO G;

Z=Z@MT[,J];END;MAT[,I]=Z;END;

MINVEC=FLOOR(XR);PSI=LVEC*MUVEC`;

PVEC=J(NROW(MAT),1,0);NMAT=J(NROW(MAT),G,0);

DO I=1 TO NROW(MAT);

NVEC=MINVEC+MAT[I,];NMAT[I,]=NVEC;

NT=SUM(NVEC);

VARPSI=(LVEC##2)*(VARVEC/NVEC)`;

DELTA=PSI/SQRT(VARPSI);DF=NT-G;DFVEC=NVEC-1;

KV=(LVEC##2)#VARVEC/NVEC;V1=SUM(KV)##2;

V2=SUM((KV##2)/(NVEC-1));DFAP=V1/V2;CRIT=TINV(1-ALPHA/2,DFAP);

APP=CDF('T',-CRIT,DFAP,DELTA)+SDF('T',CRIT,DFAP,DELTA);

PVEC[I,1]=APP;

END;

TVEC=NMAT*CVEC`;

LOC=LOC(PVEC>=POWER);

N2MAT=NMAT[LOC,];P2VEC=PVEC[LOC,];T2VEC=TVEC[LOC,];

T2MIN=T2VEC[><,];MINIVEC=LOC(T2VEC=T2MIN);

N2MINMAT=N2MAT[MINIVEC,];

P2MINVEC=P2VEC[MINIVEC,1];

T2MINVEC=T2VEC[MINIVEC,1];

POMAXMIN=P2MINVEC[<>,1];MAXMINI=P2MINVEC[<:>,1];

NMAXMIN=N2MINMAT[MAXMINI,];TOTALN=SUM(NMAXMIN);

OPTIMALN=NMAXMIN;APOWER=POMAXMIN;TOTALCOST=T2MIN;

PRINT OPTIMALN APOWER[FORMAT=7.4] TOTALCOST TOTALN;

QUIT;
